# Buffalo Academy of Medicine

**Published:** 1899-07

**Authors:** Thomas F. Dwyer


### Society Proceedings.

### BUFFALO ACADEMY OF MEDICINE.

Reported by THOMAS F. DWYER, M. D., Secretary.

ANNUAL meeting, held at the Academy parlors, Palace Arcade, Tuesday evening, June 13, 1899.

The president, Dr. Roswell Park, called the meeting to order at 9 o'clock p. m. The minutes of the last annual meeting were read and approved. The secretary of the academy and council then presented the following report which, on motion, was received and ordered spread on the minutes.

### ANNUAL REPORT OF THE SECRETARY.

*To the Academy of Medicine:*

During the past fiscal year fourteen applications for membership have been received and favorably acted upon, of which thirteen were resident fellows and one non-resident fellow. Two resignations have been received and accepted and two members have been dropped for non-payment of dues. The academy has been very fortunate in that its membership has not been decreased by death since the last annual meeting.

Article XIII., section 3, of the by-laws was amended to read: "Any fellow failing to pay his dues for two years shall forfeit his membership."

The plan of preparing a program during the summer for the entire session was so successful last year that it was repeated for the session of 1898-99 by vote of the council and a copy sent to the members of the academy.

Two special meetings were called during the year. The first on July 11, 1898, to listen to a paper upon The production of diastase and of an alcoholic ferment from fungi, by Dr. Jokichi Takamine, formerly of Tokyo University, Tokyo, Japan. The other was called on May 25, 1899, for the purpose of taking proper steps to secure the meeting of the American Medical Association in Buffalo, in June, 1901 (Pan-American year).

Resolutions were adopted extending a hearty invitation to the American Medical Association to hold its meeting here in June, 1901, and a committee of seven was appointed to represent the academy at the meeting of the Association at Columbus.

Four regular meetings were held and entertained in turn by the sections of the academy: Section on obstetrics and gynecology, June 28, 1898, Discussion and diagnosis of suppurative forms of intrapelvic disease and surgical management, Dr. Joseph Price, Philadelphia, Pa. Section on Medicine, September 13, 1898, Chronic joint affections, commonly called rheumatism, Dr. Charles G. Stockton; Rheumatism of childhood, Dr. Benjamin G. Long. Section on surgery, December 6, 1898, Curability of cancer, Dr. N. Jacobson, professor of clinical surgery University of Syracuse; Colles's fracture, Dr. Edward M. Dooley. Section on pathology, March 21, 1899, Tuberculous infections in the walls of the blood-vessels and the production of miliary tuberculosis, Dr. H. R. Gaylord.

The meetings of the academy have been fairly well attended during the past year. At the June meeting 56 were present; September meeting, 41; December meeting, 49, and the March meeting, 36, an average attendance of 45½. The average time occupied by each meeting was one hour and forty-five minutes. The council of the academy held six meetings, with an average attendance of nine members. Respectfully submitted.

THOMAS F. DWYER, Secretary.

Inasmuch as no report was received from the treasurer, the president announced that the annual report would be requested for the quarterly meeting, June 27, 1899, and then referred to an auditing committee.

Reports were received from the secretaries of the sections on medicine, surgery, obstetrics and pathology, and on motion were received and ordered filed.

Dr. DeLancey Rochester, on behalf of the tellers, reported the result of the election as follows: President, Dr. Matthew D. Mann; secretary, Dr. Thomas F. Dwyer; treasurer, Dr. Eugene A. Smith; trustee, Dr. Stephen Y. Howell.

An announcement was read from Dr. Eli H. Long, chairman of the section on medicine, that at the quarterly meeting of the academy in September a paper would be read by Dr. D. R. Brown, of Chicago, Ill., on The medical aspect of crime.

The retiring president, Dr. Roswell Park, then read his annual address, the subject of which was History of organo-therapy.

Dr. Park also thanked the officers and members of the academy for their very able assistance during the past year.

The installation of officers being in order, Dr. Matthew D. Mann, the newly elected president, was escorted to the chair and presented to the academy by Dr. Park. Dr. Mann thanked the members of the academy for this confidence in electing him to this high office. He realised that the standard was very high during the administration of his predecessor, but he hoped that this year we would reach an even higher one, as the academy should advance from year to year. Some arrangements should be made, he said, to publish the proceedings in the BUFFALO MEDICAL JOURNAL, as this would increase the interest in the academy and would greatly extend its usefulness. He also stated that the medical faculty of the University of Buffalo had directed him to tender to the academy the use of the alumni hall, including the stereopticon, for the meetings of the academy, free of expense.

It was moved and seconded that the matter of change of meeting-place be referred to the council and that they be requested to report, if possible, at the next quarterly meeting, and that notice be sent to the members when action would be taken by the academy. Carried.

Dr. J. W. GROSVENOR moved that a vote of thanks be tendered the retiring officers for their efficient work of the past year. Carried.

Attendance, 40. Adjourned at 10.10 p m.

